# Prevalence of Methicillin-Resistant Staphylococcus aureus and Classical Enterotoxin Genes Among Sudanese Food Handlers

**DOI:** 10.7759/cureus.12289

**Published:** 2020-12-25

**Authors:** Omar B Ahmed

**Affiliations:** 1 Department of Environmental and Health Research, Umm Al-Qura University, Makkah, SAU

**Keywords:** s. aureus, food handlers, enterotoxins, mrsa, mssa, sudan

## Abstract

Food handlers who carry enterotoxin-producing *Staphylococcus aureus* could become potential reservoirs of Staphylococcal food poisoning. The study is a cross-sectional one aimed to determine the prevalence of methicillin-resistant *S. aureus* (MRSA) and staphylococcal enterotoxins from randomly selected food handlers in Al Jazirah state, Sudan. Culture swabs were collected from the hands and nasals of food handlers (2016-2018). Identification of *S. aureus* was done on the basis of conventional laboratory tests. All *S. aureus* isolates were screened for MRSA and staphylococcal enterotoxin (SE) genes by polymerase chain reaction. The *S. aureus* strains were isolated from 25% of the collected culture swabs of which 42% were confirmed as MRSA. The existence of one or more of enterotoxin genes was confirmed in 34.4% of the isolated *S. aureus* strains. The combined staphylococcal enterotoxin genes were found in 9.6% of the isolates. The SE genes among MRSA strains (61.5%) were found to be higher than methicillin-sensitive *S. aureus* strains (14.8%). The most frequent staphylococcal enterotoxin genes were SEA (19.4%) followed by the SEB (8.6%), SEC (4.3%), and SED (2.1%). The carriage rate of MRSA strains demonstrated a higher rate of staphylococcal enterotoxins genes than methicillin-sensitive *S. aureus*. There is an increasing prevalence of MRSA compared with the previous rates and staphylococcal enterotoxin genes among Sudanese food handlers, which is a serious problem for public health.

## Introduction

Foodborne diseases (FBDs), which involve a wide spectrum of illnesses caused by either microbial (bacteria, viruses, or parasitic) or chemical contamination of food, now have become a global health problem. It is estimated that more than 100 million people of the population in the Eastern Mediterranean region suffer from these diseases, whereas two million deaths in developing countries occurred annually [1,2]. In Sudan, a large number of consumers suffer from FBDs, some become ill, and some die from them. The increase in FBDs in Sudan requires a rapid and effective response. Staphylococcal food poisoning (SFP) is a common FBD which is known as an intoxication due to exposure to staphylococcal enterotoxins (SEs) [3]. SEs are considered exotoxins, insensitive to heat, irradiation produced by *Staphylococcus aureus*, and of low molecular weight [4]. The prevention of SFP has an important social and economic value in Sudan. The disease could influence people working days and productivity of the workers, in addition to the hospital expenses, and financial losses in food industries, catering companies, and restaurants [5]. The known types of SEs have reached 22 in number; half of this number has emetic action [5,6]. The classical SEs were designated with consecutive letters of the alphabet, from SEA to SEE, which are involved in 95% of SFP outbreaks [7]. Food handlers may carry enterotoxin-producing *S. aureus* in their hands and secretions, hence they could become a potential source of SFP through direct contact or respiratory secretions. Methicillin-resistant *S. aureus* (MRSA) have emerged from *S. aureus* through the staphylococcal cassette chromosome mec, (carrying the mecA gene) responsible for resistance to antibiotics such as methicillin and penicillin [8]. This study aimed to determine the prevalence of MRSA and SEs among *S. aureus* isolated from food handlers in Al Jazirah state, Sudan.

## Materials and methods

Collection of the samples

This study was a descriptive qualitative cross-sectional laboratory-based study conducted in Al Jazirah state, Sudan, from July 2016 to July 2018. A total number of 372 swab specimens were collected from the nasal and hands (interdigital region, index fingers, thumbs, and palms of both right and left hands) of 186 food handlers who were working in different restaurants in different localities of Al Jazirah state. Valid consent was obtained from each subject under the study.

Bacterial isolation and identification

The swabs were immediately placed in a bacterial transport medium. They were directly inoculated onto blood agar, MacConkey agar (Oxoid, Cambridge, UK), and were incubated aerobically at 37°C for 18-24 hours. Identification of *S. aureus* was done on the basis of conventional laboratory tests. After Gram stain, colonies of Gram positive cocci were tested with catalase, hemolysis on blood agar, coagulase, DNase tests in addition to culture properties on mannitol salt agar medium.

MRSA and SEs screening

For DNA extraction, bacteria were collected and pelleted after culturing in nutrient broth. The DNA was extracted by microwave lysis method, briefly, bacteria were collected, pelleted after culturing in nutrient broth, then washed and resuspended in 10% SDS. The suspensions were then incubated for 30 minutes at 65°C. The pellets were centrifuged, the supernatants were removed, and the isolates were placed in a microwave oven and heated twice for one minute. The DNA was extracted by using the microwave method and phenol/chloroform/isoamyl alcohol method [9]. An aliquot of the DNA was electrophoresed on 1% agarose gel to ensure the purity of extracted DNA. The samples were then screened for mecA [10] and the SE genes SEA, SEB, SEC, SED, and SEE by polymerase chain reaction (PCR), with specific primers (Table 1) [10,11,12]. The PCR reaction mixture (25 μl) contained 5 μl of DNA template, 1 µl (100 pmol) of each primer, and 25 µl of Taq PCR Master Mix polymerase (Qiagen, Germantown, MD, USA). Amplification conditions were as follows: initial denaturation for five minutes at 94ºC, and then 30 cycles, each cycle consisting of denaturation at 94ºC for two minutes and extension at 72ºC for one minute. Annealing temperatures used for each step are shown in Table 1. Final extension was performed at 72ºC for five minutes. The PCR products were analyzed and visualized by electrophoresis in 1.5% agarose gel and stained with ethidium bromide (2 μg) and were viewed under UVP BioDoct It Imaging System (Analytik Jena, Jena, Germany).

**Table 1:** Primers of the genes used in the study SEs = staphylococcal enterotoxins

SEs	Sequence	Size	Temp	Target	Reference
mecA-P4	TCCAGATTACAACTTCACCAGG	162	53	mecA	[10]
mecA-P7	CCACTTCATATCTTGTAACG				
SEA-1	TTGGAAACGGTTAAAACGAA	120	50	SEA	[11]
SEA-2	GAACCTTCCCATCAAAAACA				
SEB-1	TCGCATCAAACTGACAAACG	478	50	SEB	[11]
SEB-2	GCAGGTACTCTATAAGTGCC				
SEC-1	GACATAAAAGCTAGGAATTT	257	50	SEC	[11]
SEC-2	AAATCGGATTAACATTATCC				
SED-1	CTAGTTTGGTAATATCTCCT	317	50	SED	[11]
SED-2	TAATGCTATATCTTATAGGG				
SEE-1	TAGATAAAGTTAAAACAAGC	170	50	SEE	[12]
SEE-2	TAACTTACCGTGGACCCTTC				

Statistical analysis

Statistical analysis was done using Statistical Package for Social Sciences (SPSS) version 25 software (IBM Corp., Armonk, NY, USA). The chi-square test was used to compare frequency distribution of SE genes among MRSA and methicillin-sensitive *S. aureus* (MSSA) isolates (p < 0.05 was considered statistically significant).

## Results

**Figure 1:**
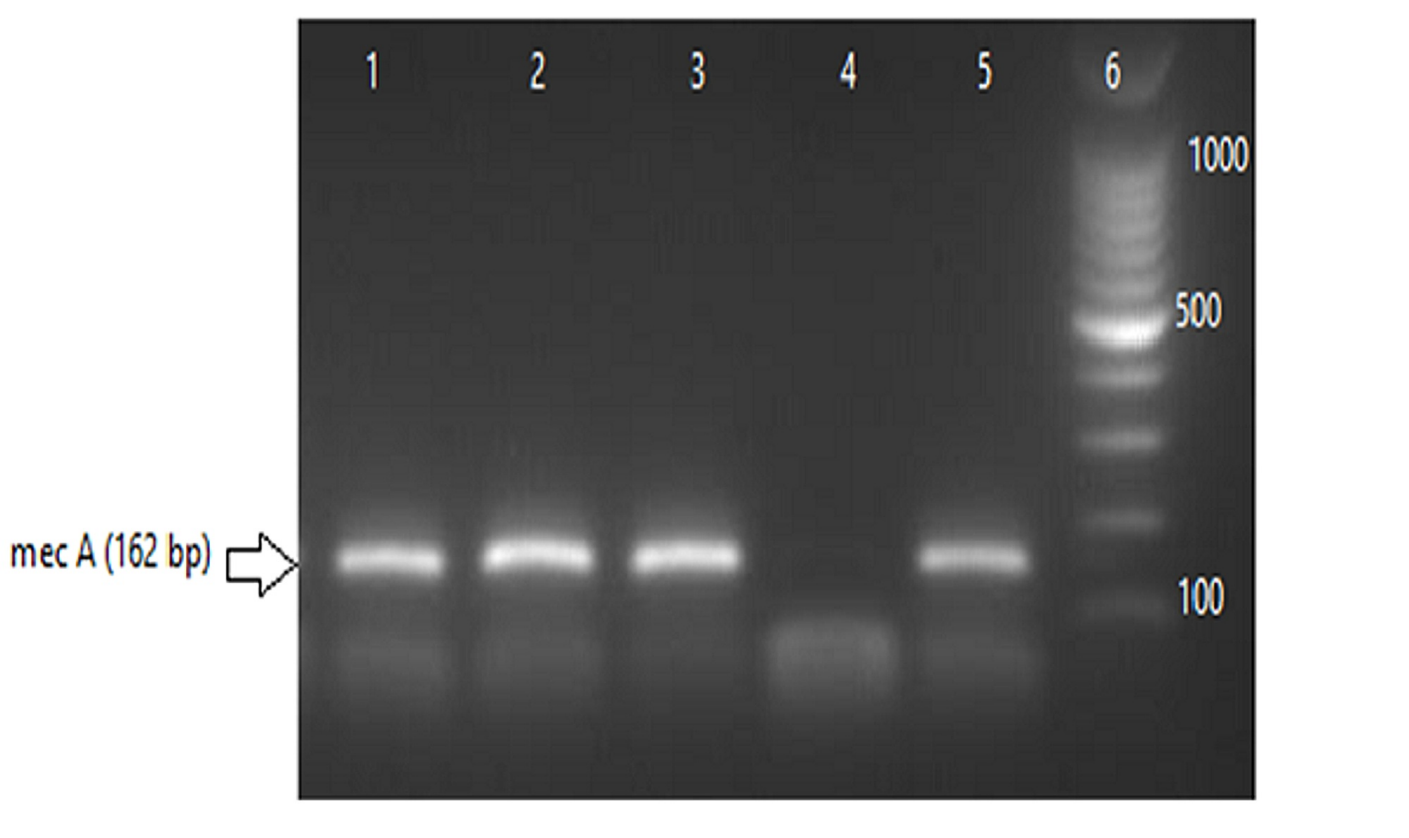
Detection of mecA gene on 2% agarose gel electrophoresis.

**Figure 2:**
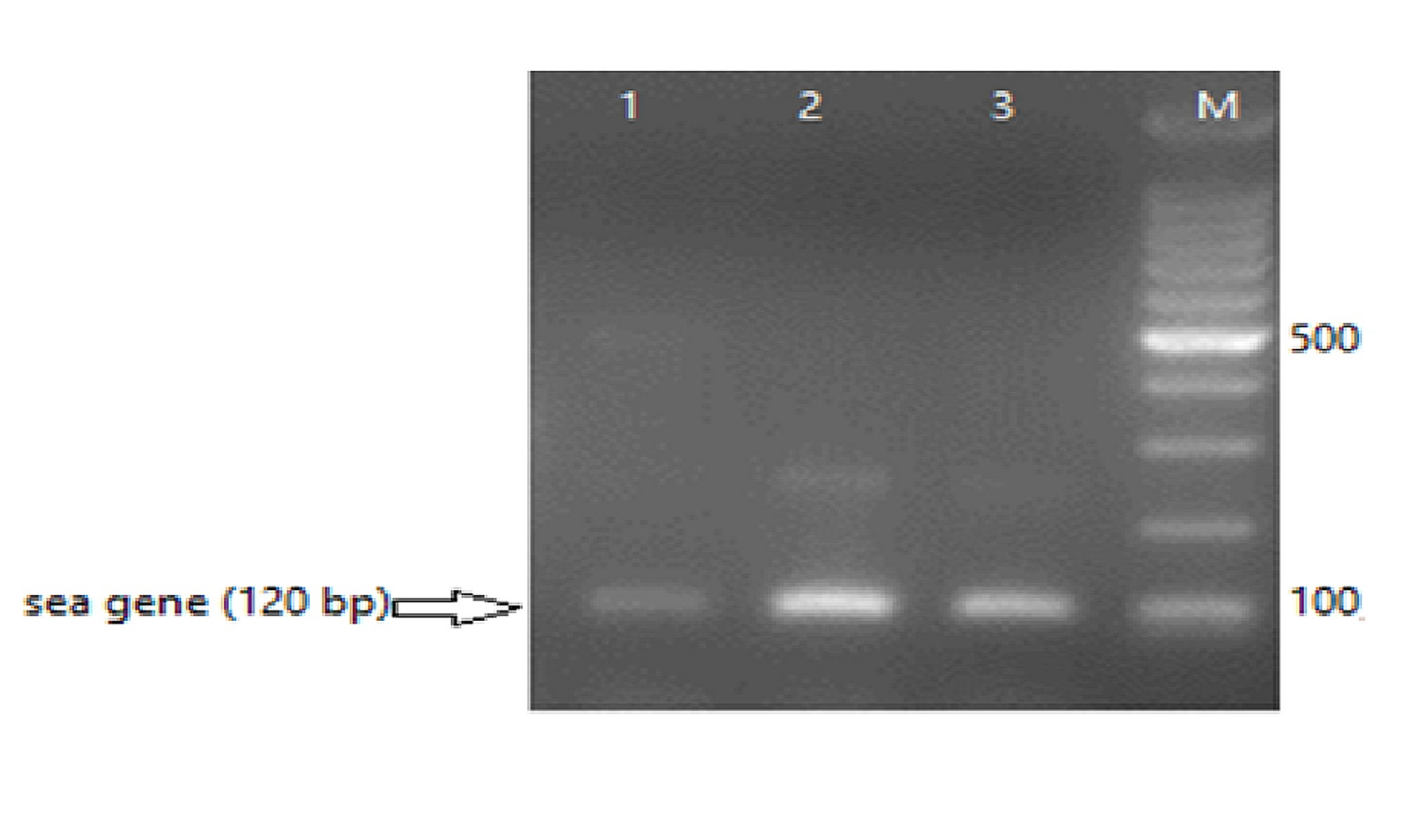
Detection of SEA gene on 2% agarose gel electrophoresis, lane M: 100-bp DNA ladder.

**Figure 3:**
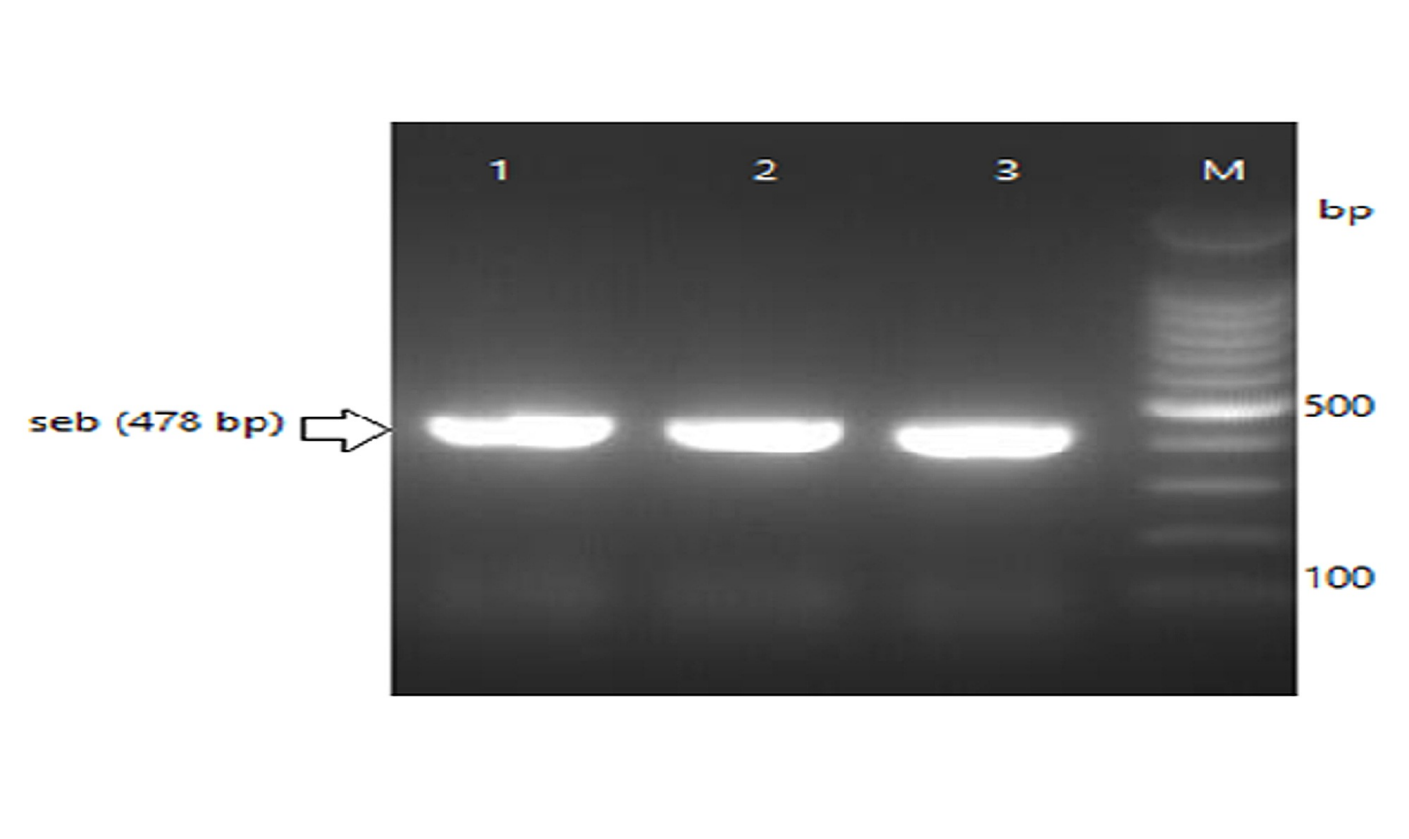
Detection of SEB genes on 2% agarose gel electrophoresis, lane M: 100-bp DNA ladder.

**Figure 4:**
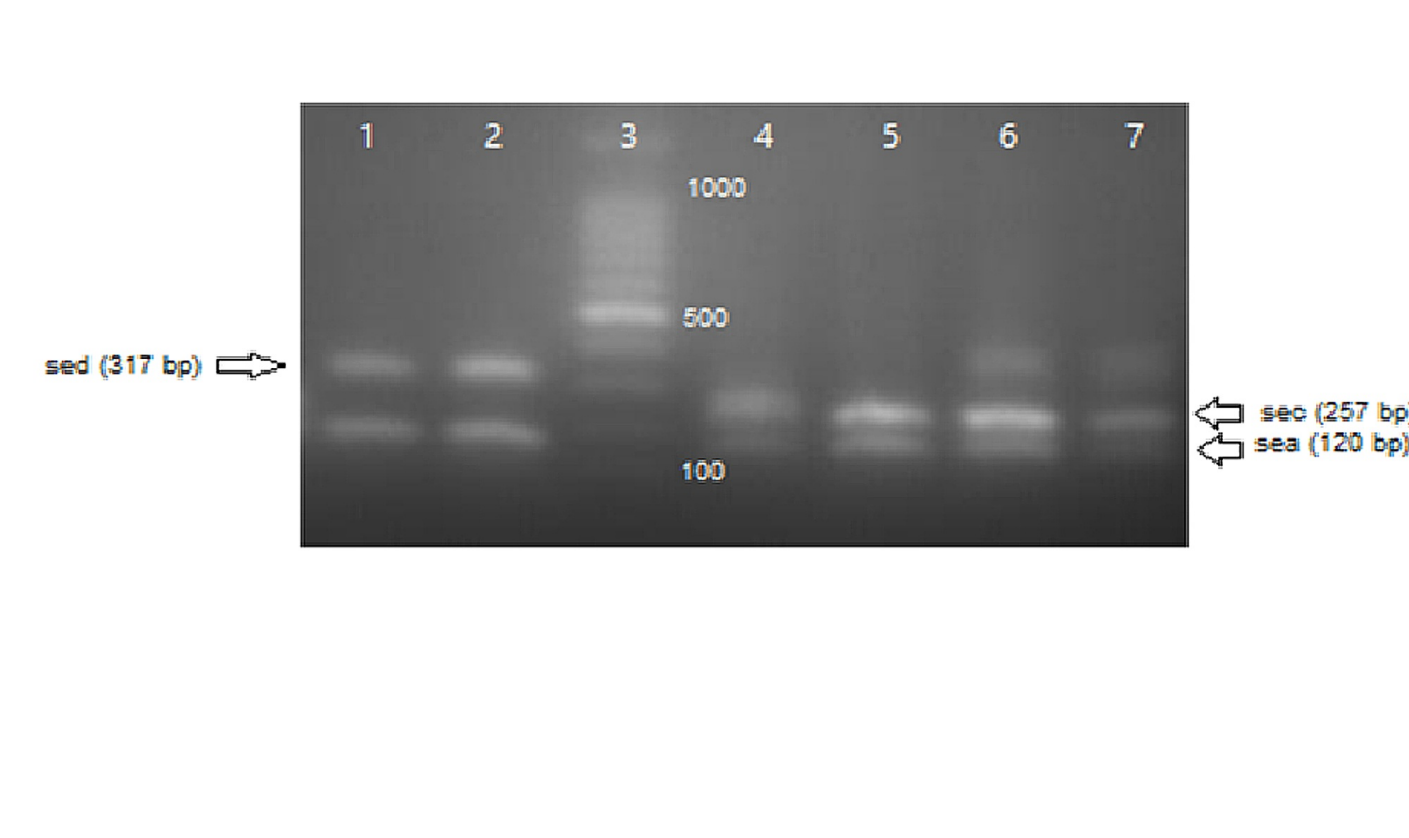
Coexistence enterotoxin genes on 2% agarose gel electrophoresis, lane M: 100-bp DNA ladder.

A total number of 372 swab specimens were collected from food handlers working in kitchens or cafeterias in Al Jazeera state, Sudan. The number of isolated *S. aureus* strains was 93 (25%) as shown in Table 2. PCR results confirmed the existence of one or more of enterotoxin genes in 32 (34.4%) out of total *S. aureus* strains (Table 2, Table 3). Overall, the total number of MRSA confirmed by PCR (Figure 1) was 39 (42%). Of these MRSA strains, 24 (61.5%) harbored at least one enterotoxin gene (Table 3). SEA was the highest detected gene (19.4%) followed by SEB (8.6%), SEC (4.3%), SED (2.1%), and SEE (0%) (Table 2). The combination SE(A+C) genes were detected in 4.3% out of the total 93 of *S. aureus* strains followed by the SE (A+B) (3.2%), and SE (A+D) (2.1%) as shown in Table 2 and Figures 2-3. The co-existence of more than one enterotoxin gene was detected in nine (9.6%) out of the total 93 of *S. aureus* strains as shown in Table 2 and Figures 3-4. Significantly, MRSA strains showed a higher prevalence (61.5%) of SE genes than MSSA (14.8%) (P < 0.05).

**Table 2:** Frequency of the SE genes in the isolated S. aureus strains. SE = staphylococcal enterotoxin

SE gene	No (%)	Combined SE genes	No (%)
SEA	18 (19.4%)	SE (A+B)	3 (3.2%)
SEB	8 (8.6%)	SE (A+C)	4 (4.3%)
SEC	4 (4.3%)	SE (A+D)	2 (2.1%)
SED	2 (2.1%)		
SEE	0 (0%)		
Total	32 (34.4%)	Total	9 (9.6%)

**Table 3:** Distribution of the SE genes among the isolated MRSA and MSSA strains. SE = staphylococcal enterotoxin, MRSA = Methicillin-resistant S. aureus, MSSA = methicillin-sensitive S. aureus

SE genes	MRSA	MSSA	Total
SEA	14 (15.1%)	4 (4.3%)	18 (19.4%)
SEB	6 (6.4%)	2 (2.1%)	8 (8.6%)
SEC	2 (2.1%)	2 (2.1%)	4 (4.3%)
SED	2 (2.1%)	0 (0%)	2 (2.1%)
SEE	0 (0%)	0 (0%)	0 (0%)
Total	24 (61.5%)	8 (14.8%)	32 (34.4%)

## Discussion

Food handlers could be a potential source of foodborne illness or food poisoning which spread due to inadequate personal hygiene or from eating contaminated, spoiled, or toxic food. Bacteria is the most prevalent cause of food poisoning. The present study was undertaken to investigate the distribution of classical SE genes in *S. aureus* isolated from food handlers in Al Jazirah state, Sudan. The results showed that 93 (25%) of the food handlers were carriers of *S. aureus*. The reported prevalence of *S. aureus* on the hands of food handlers is variable. Previous studies in Sudan reported that 21.6% of food handlers were found harboring *S. aureus* [13]. Studies from different countries have indicated that food handlers have a 20-30% carriage rate of *S. aureus* [14-17], while others reported higher results (37.5%) [18]. Also the study showed that 42% of *S. aureus* carried the mecA gene (MRSA). This finding is higher than Sezer et al., 2015 [19] (28.9%), and lower than Loeto et al., 2007 (57.5%) in Botswana [20]. The high prevalence of MRSA could be due to the extensive usage of antibiotics in the Al Jazirah community without prescription. The carriage of MRSA among food handlers could lead to development of multidrug-resistant bacteria and would become an important risk factor in the contamination of food as well as a source of staphylococcal human transmission [21,22]. The present study described classical SEs from SEA to SEE. PCR results confirmed the existence of enterotoxin genes in 34.4% of *S. aureus* isolates which is higher than Loeto et al., 2007 (21%) [20], and lower than Figueroa et al. 2002 [23] (54%) and Udo et al., 2009 [16] (71%), respectively. The MRSA strains in the present study demonstrated a higher prevalence (61.5%) of SE genes which is significantly higher than MSSA (14.8%) (P < 0.05). This may be due to the possible handling of contaminated food and multiple infection with SFP among food handlers [24]. The present study showed that a combination of more than one enterotoxin gene was found in 9.6% of *S. aureus* isolates. The most frequent gene combination was SEA with SEC (4.3%), similarly Fooladvand et al., 2019 [24] detected 40.8%,14.7%, and 1.9% combination of two, three, or four SEs genes, respectively. Also, Udo et al., 2009 [16] demonstrated that 38%, 20%, and 7% of *S. aureus* isolates contained two, three, and four SE genes respectively. SEA was the most frequently detected gene (19.4%) followed by SEB (8.6%), SEC (4.3%), SED (2.1%), and SEE (0%) genes. Worldwide, SEA has been described as the most common by many other authors [18,25,26]. This is in contrast to other studies conducted where the predominant SE gene was toxin SEB [27,28]. Worldwide, other enterotoxin genes have been described by many authors as in Poland SEC (17.5%), SED (5%), and SEE (0%) [29], also in the Netherlands SEC (7.5%), SED (1.9%) and SEE (0%) [26]. Food handlers carrying enterotoxin-producing *S. aureus* can contaminate food, and hence become a potential risk of food poisoning.

## Conclusions

In conclusion, the prevalence of *S. aureus* in food handlers (25%) is similar to the rate reported worldwide. The increasing prevalence of MRSA in food handlers in Sudan is a serious problem for public health. The high prevalence of SE genes indicates a potential risk of food poisoning. The MRSA strains in the present study demonstrated higher prevalence (61.5%) of SE genes which is significantly higher than MSSA in Sudanese food handlers.
